# Direct medical costs of fall-related injuries in elderly patients presenting to a tertiary hospital: A descriptive study

**DOI:** 10.12669/pjms.42.6.15680

**Published:** 2026-06

**Authors:** Omer Faruk Tekin, Cagdas Sertkaya, Muammer Yilmaz, Inci Arikan

**Affiliations:** 1 Omer Faruk Tekin, Assistant Professor, Department of Public Health, Faculty of Medicine, Kütahya Health Sciences University, Kütahya, Türkiye; 2 Cagdas Sertkaya, Research assistant, Department of Public Health, Faculty of Medicine, Kütahya Health Sciences University, Kütahya, Türkiye; 3 Muammer Yilmaz, Associate Professor, Department of Public Health, Faculty of Medicine, Kütahya Health Sciences University, Kütahya, Türkiye; 4 Inci Arikan, Professor, Department of Public Health, Faculty of Medicine, Kütahya Health Sciences University, Kütahya, Türkiye

**Keywords:** Aged, Accidental Falls, Direct Service Costs, Health Care Costs, Tertiary Hospital

## Abstract

**Background & Objectives:**

Fall-related injuries are a leading cause of injury and injury-related death among elderly people aged 65 and over and are a significant public health problem. Determining the economic burden of elderly people who are admitted to hospital due to falls will guide the development of policies and plans regarding healthcare delivery. This study aimed to calculate the direct medical costs associated with the treatment of fall-related injuries in individuals aged 65 and over who presented to a tertiary hospital due to falls.

**Methodology:**

This descriptive study was conducted in Kütahya, Türkiye, between September 15, 2022 to December 15, 2022. The study data were obtained from the data system of Kütahya Health Sciences University Evliya Celebi Training and Research Hospital. The study population consisted of individuals over the age of 65 diagnosed with falls between July 1, 2021, and June 30, 2022. Hospital records were examined, and the unit prices of the medical procedures performed, including subsequent treatments related to the fall, were obtained according to the unit prices defined in the Social Security Institution’s reimbursement tariff. Direct medical costs were categorized accordingly. All costs were calculated in Turkish Lira (TRY) and converted to United States Dollars (USD) using the average exchange rate for the study period (1 USD = 12.60 TRY). Data analysis was conducted using the Jamovi v2.3 software.

**Results:**

An analysis of the distribution of fall-related costs revealed that the highest costs were associated with intensive care (101,778 USD; 27.3%), medical equipment (83,318 USD; 22.4%), surgery (44,807 USD; 12.0%), medication (43,669 USD; 11.7%), and medical services (21,388 USD; 5.7%). The total cost of falls in the study group amounted to 372,563 USD, with inpatients accounting for 332,854 USD and outpatients for 39,710 USD.

**Conclusions:**

Falls impose a significant financial burden on healthcare systems, particularly due to high-cost components such as intensive care. Effective preventive interventions can reduce these costs while improving the health and well-being of older adults.

## INTRODUCTION

With the increase in the world population and the extension of human life, the elderly population in the countries is also increasing rapidly. In 2024, one in ten individuals was aged 65 and over, while by 2080, it is projected that one in three individuals will be elderly.^1^ With aging, age-related risks also increase. One of these risks is fall-related injuries. Fall-related injuries are a leading cause of injury and injury-related death among adults aged 65 and over and are a significant public health problem.^2^ While most fall-related injuries result in minor injuries such as abrasions, bruises and sprains, injuries severe enough to require surgery and intensive care can also ocur.^3^

Socioeconomic effects of injuries resulting from falls in older people are also evident. The resulting direct and indirect costs constitute a significant burden on health systems. Direct medical costs refer to the money spent directly by patients or the government to treat falls, while indirect costs refer to the financial losses incurred by the patient and caregiver due to loss of labor.^4^ There are numerous studies on the economic costs of falls in the elderly, especially in the USA.^5^-^9^ Although studies on this subject have begun to be conducted in Turkey in recent years, more evidence is needed to clearly present the picture. Determining the economic burden of elderly people who are admitted to hospital due to falls will guide the development of policies and plans regarding health service delivery.

This study aimed to calculate the direct medical costs of expenses incurred by the hospital for the treatment of fall-related injuries of people aged 65 and over who applied to a training and research hospital with complaints of falls.

## METHODOLOGY

**Fig.1:**
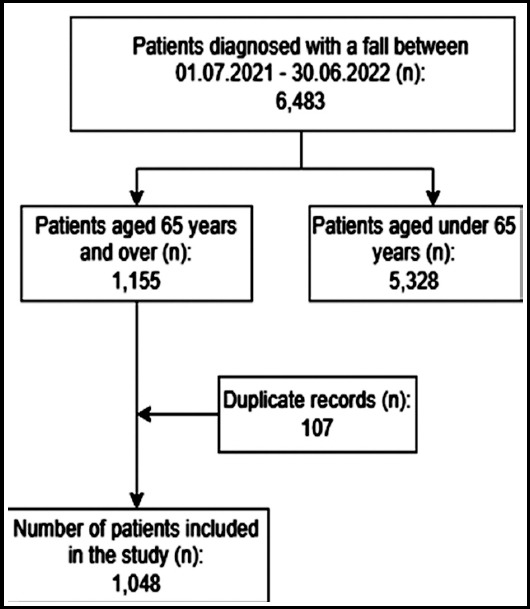
Work flow algorithm.

This is a descriptive study conducted in Kütahya, Türkiye, between September 15, 2022 to December 15, 2022. Since this was a retrospective study based on the review of patient medical records, informed consent was not required. The population of Kütahya province, located in the west of Turkey, is 580,701 (81,117 (13.96%) people aged over 65). The population of the central district where the study was conducted is 282,243 people and there is one public hospital and two private hospitals. The study data were obtained from the data system of Kütahya Health Sciences University Evliya Celebi Training and Research Hospital, which has a capacity of 950 beds and is the only public hospital in the region. The universe of the study consists of individuals over the age of 65 who were diagnosed with falls between 01.07.2021 and 30.06.2022. In the data collection phase of the study, firstly, a list of patients over the age of 65 who were diagnosed with falls (ICD-10 codes W00-W19) on the relevant dates was obtained and duplicate records were eliminated (Fig.1). In the second stage, the hospital applications of these people were examined and the unit prices of the procedures performed, including subsequent applications related to the fall situation, were obtained according to the unit prices defined in the Social Security Institution’s reimbursement tariff.

Direct medical costs were grouped as outpatient clinic, medicine, medical supplies, medical services, laboratory tests, imaging tests, service, intensive care, surgery, blood product, prosthesis and home care fees.

### Ethical approval and Consent:

Ethics committee approval and hospital chief physician permission were obtained (Ethics committee decision received from Kütahya Health Sciences University dated 14.09.2022 and numbered 2022/09-14). The patient consent was waived due the retrospective design.

### Statistical analysis:

The data of the study were evaluated with the Jamovi v2.3 package program. Descriptive statistics were given as mean and standard deviation for numerical variables and as number and percentage for categorical variables. Unit cost prices were presented in Turkish Lira (TRY) and American Dollar (USD). Since the USD showed a fluctuating trend above the TRY in the Turkish economy in the period of 2021-2022, which is the period in which the study was conducted, the exchange rate, which is the average USD/TRY price in the one-year period in which the study was conducted, was accepted as 1 USD = 12.60 TRY.

## RESULTS

The study was completed with data from 1048 individuals, 59.6% of whom were female (n=625) and 40.4% male (n=423). The age groups of the participants were 45.3% (n=475) 65-74 years old, 36.5% (n=383) 75-84 years old, and 18.2% (n=190) 85 years old and over. According to the seasons in which the falls occurred, 29.8% (n=312) were in summer, 25.1% (n=263) in autumn, 23.1% (n=242) in spring and 22% (n=231) in winter. Of the participants, 24.2% (n=254) required hospitalization, 16.4% (n=171) required intensive care, 14.2% (n=148) required surgery due to a fall, and 15.3% (n=160) required home health care after a fall (Table-I).

**Table-I:** Sociodemographic characteristics of the participants.

	No.	Percentage
***Gender***		
Male	423	40.4%
Female	625	59.6%
***Age Group***		
65-74	475	45.3%
75-84	383	36.5%
85 and above	190	18.2%
***Diagnosis Code***		
Fall. unspecified	856	81.6%
Fall from same level	168	16.0%
Fall from stairs and steps	10	0.9%
Fall from one level to another	10	1.0%
Fall from tree	3	1.0%
Fall on ice and snow	1	0.1%
***Season***		
Spring	242	23.1%
Summer	312	29.8%
Autumn	263	25.1%
Winter	231	22.0%
***Hospitalization status***		
No	794	75.8%
Yes	254	24.2%
***Intensive care admission***		
No	877	83.6%
Yes	171	16.4%
***Fall-related surgery***		
No	900	85.8%
Yes	148	14.2%
No	888	84.7%
Yes	160	15.3%
	*Mean ± SD*	*Min-Max*
Age	76.69 ± 7.69	65 - 102
Length of Hospitalization (days) (n=254)	10.11 ± 7.38	1 - 50

When the distribution of fall costs of the study group was examined, the largest costs were intensive care cost (1,282,399 TRY; 101,778 USD; 27.3%), medical equipment cost (1,049,812 TRY; 83,318 USD; 22.4%), surgery cost (564,572 TRY; 44,807 USD; 12.0%), medication cost (550,229 TRY; 43,669 USD; 11.7%) and medical service cost (269,486 TRY; 21,388 USD; 5.7%), respectively (Table-II).

**Table-II:** Distribution of costs.

Costs	Mean (₺($))	Standard Deviation (₺($))	Total (₺($))	Percentage (%)
Polyclinic	96.4 (7.6)	276.2 (21.9)	100,990 (8,015)	2.2
Medicine	525 (41.7)	2,622 (208.2)	550,229 (43,669)	11.7
Medical supplies	1,001 (79.5)	4,273 (339.1)	1,049,812 (83,318)	22.4
Medical services	257.1 (20.4)	864.7 (68.6)	269,486 (21,388)	5.7
Laboratory	134.9 (10.7)	352.3 (28)	141,397 (11,222)	3.0
Imaging	164.5 (13.1)	199 (15.8)	172,440 (13,686)	3.7
Service inpatient	105.7 (8.4)	451.5 (35.8)	110,796 (8,793)	2.4
Intensive care inpatient	1,223 (97.1)	7,393 (586.8)	1,282,399 (101,778)	27.3
Surgery	538.7 (42.8)	1,876 (149)	564,572 (44,807)	12.0
Blood product	195.3 (15.5)	729.1 (57.9)	204,654 (16,242)	4.4
Prosthesis	183.6 (14.6)	687.9 (54.6)	192,383 (15,269)	4.1
Home care services	53 (4.2)	293 (23.3)	55,545 (4,408)	1.2
Total	4,479 (355.5)	12,266 (973.5)	4,694,299 (372,563)	100

₺: Turkish Lira, $: USD.

**Fig.2:**
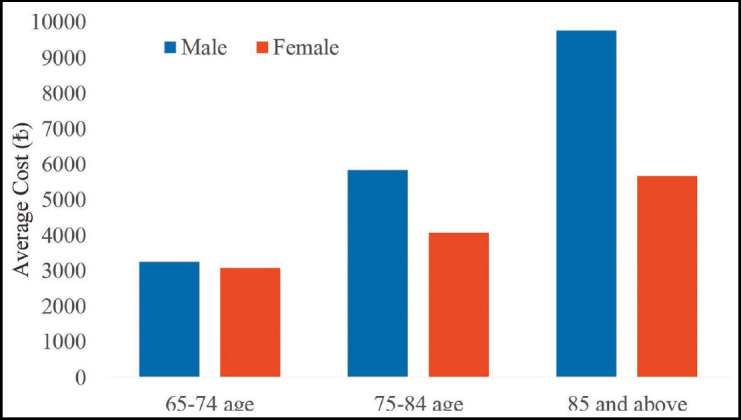
Distribution of average costs per person by gender.

The total cost of falls in the study group was 4,694,299 TRY (372,563 USD), with a total cost of 4,193,958 TRY (332,854 USD) for inpatients and 500,340 TRY (39,710 USD) for outpatients. Mean and total cost distributions by age and gender are presented in Table-III and Fig.2.

**Table-III:** Distribution of inpatient and outpatient costs by gender and age (American Dollar).

	Inpatient			Outpatient			Total		
	N (%)	Avarege cost ($)	Total ($)	N (%)	Avarege cost ($)	Total ($)	N (%)	Avarege cost ($)	Total ($)
Sex									
Men	119 (46.9%)	1,360	161,839	304 (38.3%)	51.5	15,646	423 (40.4%)	420	177,485
Age									
65-74	46 (38.7%)	1,009	46,418	158 (52%)	39.7	6,279	204 (48.2%)	258	52,698
75-84	44 (37%)	1,368	60,193	100 (32.9%)	64.7	6,470	144 (34.0%)	463	66,663
85 and over	29 (24.4%)	1,904	55,227	46 (15.1%)	63.0	2,897	75 (17.8%)	696	58,124
Women	135 (53.1%)	1,267	171,015	490 (61.7%)	49.1	24,063	625 (59.6%)	312	195,078
Age									
65-74	44 (32.6%)	1,159	50,985	227 (46.3%)	67.2	15,243	271 (43.4%)	244	66,228
75-84	53 (39.3%)	1,342	71,104	186 (38%)	32.5	6,040	239 (38.2%)	323	77,144
85 and over	38 (28.1%)	1,287	48,926	77 (15.7%)	36.1	2,780	115 (18.4%)	450	51,706
Total	254 (100%)	1,310	332,854	794 (100%)	50.0	39,710	1,048 (100%)	355	372,563
Age									
65-74	90 (35.4%)	1,082	97,403	385 (48.5%)	55.9	21,523	475 (45.3%)	250	118,926
75-84	97 (38.2%)	1,354	131,298	286 (36.0%)	43.7	12,509	383 (36.5%)	375	149,363
85 and over	67 (26.4%)	1,554	104,155	123 (15.5%)	46.2	5,677	190 (18.2%)	578	109,830

$: USD.

While inpatient and outpatient treatment costs related to falls did not differ by gender (p>0.05), total costs were significantly higher in men than in women (p=0.023). A significant increase in total costs with age was observed among age groups (p<0.001) (Table-IV).

**Table-IV:** Comparison of Fall-Related Healthcare Costs by Gender and Age Groups.

	Inpatient		Outpatient		Total	
	Avarege cost ($)	Statistical Analysis	Avarege cost ($)	Statistical Analysis	Avarege cost ($)	Statistical Analysis
***Gender***						
Male	1,360	-0.672^a^;	51.5	-1.039 ^a^;	420	-2.272 ^a^;
Female	1,267	0.502^c^	49.1	0.299 ^c^	312	0.023 ^c^
***Age Group***						
65-74	1,082	5.125^b^;	55.9	15.688 ^b^;	250	35.918 ^b^;
75-84	1,354	0.077 ^c^	43.7	<0.001 ^c^	375	<0.001 ^c^
85 and above	1,554		46.2		578	

$: USD,

a Mann Whitnet U test Z value,

b Kruskal Wallis test H value,

c p value.

## DISCUSSION

The findings of this study indicate that women had a higher incidence of falls compared to men, and that the frequency of falls was greater in the younger-old age group than in other age groups. These findings are consistent with the literature.^10^-^14^ Alamri et al.^14^ It was determined that the elderly who applied to the hospital with a fall had an average cost of 4,479 TRY (355 USD) per patient and a total of 4,694,299 TRY (372,563 USD) per year. The average and total highest expenditure per patient was for intensive care, medical supplies, surgery and medicine. The average cost per patient increased with age. This increase was greater in men.

In health service delivery, every disease has direct and indirect costs.^15^-^17^ Falls, like other illnesses, have direct and indirect costs. Falls in older adults have a significant impact on health outcomes, as well as a financial impact on both the patient and the healthcare system.^18^ Insurance spending for trauma, including falls, in the USA has been found to be greater than annual spending for congestive heart failure, pneumonia and stroke.^19^ Studies have estimated that falls cost patients between $351 and $13,616 per patient.^20^ In the USA, direct medical costs for nonfatal falls in the elderly increased from $19 billion in 2006 to from $30.3 billion in 2012 to $31.3 billion in 2015.^8^,^21^ In this study, it was calculated that the average cost of elderly people admitted to the hospital due to falls was 4,479 TRY (355 USD) per patient and 4,694,299 TRY (372,563 USD) in total. When the studies are examined, it is seen that the information on the costs of patients who apply with falls and receive inpatient treatment is limited and outdated. In addition, some studies have obtained different results regarding cost.^8^,^18^,^20^ Since falls are preventable events, it is important to know their health effects and costs. Therefore, studies should be increased to determine the costs related to falls in the elderly accurately and up-to-date.

In this study, the factors that increased the average and total cost per patient the most were determined as intensive care admission, medical equipment used, whether surgery was performed or not, and the medications used. In this study, approximately one-fourth of the elderly who applied to the hospital with a fall were hospitalized. In the study of Kundakçı et al., this rate was found to be approximately one-fifth.^12^ Trauma, including falls, accounts for 5.6% of all hospitalizations in the USA. This rate is lower than congestive heart failure but higher than acute myocardial infarction, pneumonia, and stroke.^19^

Additionally, this study found that approximately fifteen out of a hundred elderly people who applied to the hospital with a fall complaint needed intensive care, fall-related surgery, and home health care. Intensive care admission is important because it is one of the factors that increase the cost of surgery related to falls. Intensive care costs have an important place in hospital costs. Intensive care costs constitute 30% of hospital costs. The daily intensive care cost is the highest on the first day, the daily cost decreases on the second day, and the daily cost stabilizes after the third day. In other words, even if the patient stays in the intensive care unit for a short time, it will cause high costs.^22^

In this study, the total direct cost of the elderly who applied with a fall and received inpatient treatment was 4,193,958 TRY (332,854 USD), while the cost of the elderly who received outpatient treatment was 500,340 TRY (39,710 USD). In the USA, 63% ($12 billion) of the direct costs of nonfatal falls in older adults were spent on hospitalizations, 21% ($4 billion) on emergency department services, and 16% ($3 billion) on outpatient care.^8^ It is understood from these results that patients who have serious injuries due to falls requiring hospitalization and intensive care increase costs. Therefore, taking measures to reduce the hospitalization and intensive care admission of patients who fall will reduce costs. In order to reduce costs; the number of falls of older adults should be reduced or serious injuries that may occur due to falls should be prevented.

According to this study; total cost was found to be higher in women than in men. Studies conducted in the USA also found the cost to be higher in women.^8^,^21^ In the US in 2015, the total cost for men was $8.8 billion and the total cost for women was $21.5 billion.^21^ In this study, the total average cost per patient was found to be higher in men. In male elderly patients presenting with a fall, the total average cost per person was higher for both inpatient and outpatient care. In the USA, the cost per patient was found to be higher in women, as was the total cost.^21^ According to this research; as age increases, the total average cost per person increases. A similar result was reached in the study conducted in the USA.^21^

However, in this study, this increase is more pronounced in men. In the study conducted in the USA, it is higher in women than in men.^21^ The cost may have been higher because elderly women applied with falls compared to men. However, the fact that the cost per patient is higher in men indicates that men are more likely to suffer more serious injuries as a result of falls. The reasons for the higher number of falls in women and the fact that men experience more serious injuries as a result of falls should be investigated.

This study provides a detailed breakdown of fall-related healthcare costs across multiple components (e.g., intensive care, surgery, medication, and home care) in a large sample of patients treated at a tertiary hospital. With data from a middle-income country, it also helps fill a gap in the existing literature. However, further research is needed to increase generalizability, identify long-term costs including indirect burdens, and evaluate prevention strategies and their cost-effectiveness, particularly in underrepresented regions in the literature, especially South Asia.

### Limitations:

It was conducted in a single tertiary hospital, which may limit the ability to generalize the findings to different regions or healthcare settings. The analysis focused solely on direct medical costs recorded in hospital systems; indirect costs such as loss of productivity or the burden on caregivers were not included. In addition, some clinical variables such as comorbid conditions, the severity of the fall, and pre-fall functional status were not available in the records, which may have influenced cost variations. Costs were calculated based on 2021–2022 data, a period characterized by high inflation in Türkiye; therefore, USD-based estimates may be influenced by exchange rate fluctuations and may not be directly generalizable to other periods. As the study was retrospective in nature, it does not allow for causal interpretations.

## CONCLUSION

Total treatment costs were found to be higher in males and increased significantly with age. Falls impose a considerable burden on healthcare systems, particularly due to high-cost components such as intensive care and represent a preventable public health issue. This study underscores the need for targeted intervention programs and policy initiatives aimed at reducing fall-related hospitalizations and healthcare expenditures. Implementing age-friendly environmental modifications and evidence-based fall prevention strategies can mitigate the risk of falls, thereby improving health outcomes and reducing financial strain on healthcare systems.

### Author’s Contribution:

**OFT, MY CS, IA:** Literature search, Conceptualization. Investigations. Data curation.

**OFT, CS, MY, IA:** Writing, Critical analysis and editing.

**OFT, MY, IA:** Literature review, Supervision.

All authors have read and approved the final version of the manuscript and are fully responsible for its accuracy.
